# Webspinners in Early Eocene amber from western India (Insecta, Embiodea)

**DOI:** 10.3897/zookeys.148.1712

**Published:** 2011-11-21

**Authors:** Michael S. Engel, David A. Grimaldi, Hukam Singh, Paul C. Nascimbene

**Affiliations:** 1 Division of Entomology (Paleoentomology), Natural History Museum, and Department of Ecology & Evolutionary Biology, 1501 Crestline Drive – Suite 140, University of Kansas, Lawrence, Kansas 66049-2811, USA; 2 Division of Invertebrate Zoology (Entomology), American Museum of Natural History, Central Park West at 79th Street, New York, New York 10024-5192, USA; 3 Birbal Sahni Institute of Paleobotany, 53 University Road, Lucknow 226 007, India

**Keywords:** Polyneoptera, Embioptera, Embiidina, Neoembiodea, Tertiary, taxonomy, India

## Abstract

The family Scelembiidae (Neoembiodea: Embiomorpha: Archembioidea) is recorded from Asia for the first time, based on two individuals preserved in Early Eocene amber from the Cambay Basin, western India. *Kumarembia hurleyi* Engel & Grimaldi, **gen. n. et sp. n.**, is described, figured, and distinguished from other archembioid genera. The genus shares male genitalic features with scelembiids, otherwise known from South America and Africa.

## Introduction

Embiodea are one of the more infrequently encountered and investigated orders of insects. This is unfortunate given their remarkable morphological specializations, most of which relate to the production of and life within silken galleries. For example, the probasitarsus is greatly swollen and encompasses distinctive silk glands from which the galleries are spun. The wings are unique among the flying insects for their great flexibility, permitting individuals to move in reverse through their silken tunnels, but can be made more rigid by pumping haemolymph into distinctive ‘blood sinuses’, enabling them to gain temporary rigor and permit controlled flight. Females are apterous, while males can be either fully winged or shed their wings, much like termites. Even more fascinating is that where known, all species are gregarious, living in small colonies, much like their putative relatives among the Zoraptera.

The relationship of Embiodea to other orders has been problematic, much like everything pertaining to the phylogeny of webspinners. Among the numerous competing hypotheses, those with the greatest support are a relationship to the Phasmatodea (e.g., Rähle 1970; Terry and Whiting 2005; Kjer et al. 2006; Ishiwata et al. 2011) or the Zoraptera (e.g., Engel and Grimaldi 2000; Grimaldi and Engel 2005; Yoshizawa 2007, 2011). Considerable work continues documenting the diversity of the order, with many hundreds of new species awaiting description (Ross 2000), and revising hypotheses of relationship based on this growing knowledge of the range of morphological variation observed across this fascinating group. Unfortunately, the fossil record has to date provided minimal insights toward clarifying systematic issues pertaining to embiodean evolution. This is because only nine definitive webspinner species are known from the fossil record (Table 1) and many of these are relatively modern, thereby relating more to questions of Tertiary biogeography than to higher-level branching patterns, many of which likely date from the Early Cretaceous or even Late Jurassic. Given this sparse record, any fossil webspinners are of considerable significance.

**Figure 1.**
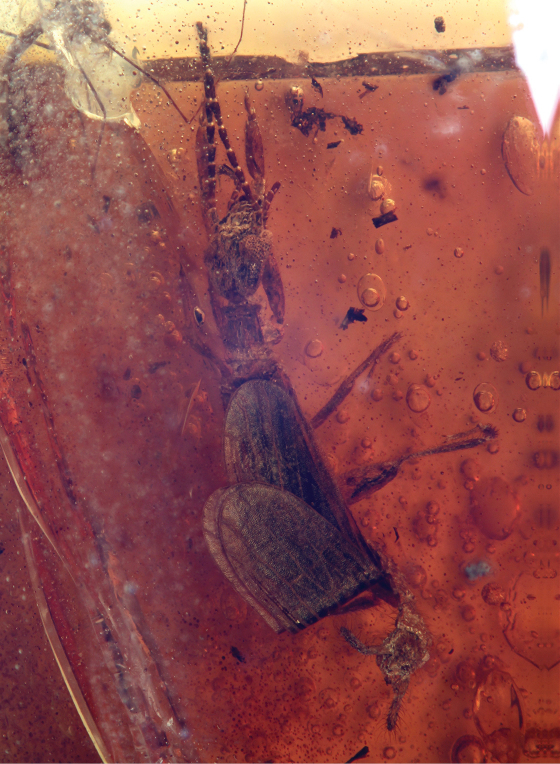
Photomicrograph of holotype male (Tad-261-A) of *Kumarembia hurleyi* Engel & Grimaldi, gen. et sp. n., in Early Eocene amber from western India. Total length of individual 5.3 mm.

**Figure 2. F2:**
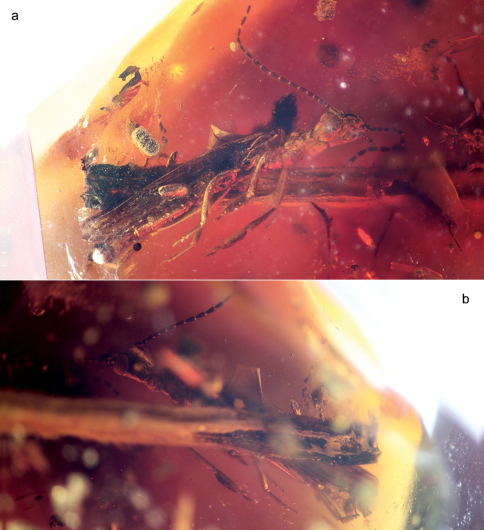
Photomicrographs of paratype male (Tad-253) of *Kumarembia hurleyi* Engel & Grimaldi, gen. et sp. n., in Early Eocene amber from western India. **A** Ventral aspect **B** Dorsal aspect. Total length of individual 5.2 mm.

Herein we provide the description of a new genus and species of fossil webspinner based on two exceptionally well preserved individuals (Fig. 1, 2) recently recovered from Early Eocene amber of the Cambay Basin in western India. These are the first fossil webspinners from Asia (Table 1) and also the first records of their family, Scelembiidae, from the Oriental Region.

**Table 1.** Described fossil webspinners (updated from Engel and Grimaldi 2006). The fossil *Clothonopsis miocenica* Hong and Wang (1987) from the Miocene of China was originally described as a clothodid but is actually a bibionid fly (Zhang 1993).

**Embiodea: Neoembiodea**		
**Teratembiidae**		
*Oligembia vetusta* Szumik, 1994	Miocene (Burdigalian)	Dominican Republic
**Anisembiidae**		
*Poinarembia rota* Ross, 2003b	Miocene (Burdigalian)	Dominican Republic
*Glyphembia amberica* Ross, 2003b	Miocene (Burdigalian)	Dominican Republic
*Glyphembia vetehae* (Szumik, 1998) Ross, 2003b	Miocene (Burdigalian)	Dominican Republic
**“Embiidae”**		
“*Embia florissantensis*” Cockerell, 1908*	Eocene-Oligocene	Colorado
*Electroembia antiqua* (Pictet, 1854) Ross, 1956	Eocene (Lutetian)	Baltic
**Scelembiidae**		
*Kumarembia hurleyi*, gen. et sp. n.	Eocene (Ypresian)	India
**Pachylembiidae: Sorellembiinae**		
*Sorellembia estherae* Engel & Grimaldi, 2006	Cretaceous (Albian)	Myanmar
**Notoligotomidae: Burmitembiinae**		
*Burmitembia venosa* Cockerell, 1919	Cretaceous (Albian)	Myanmar
*Incertae Sedis*		
**Sinembiidae**		
*Sinembia rossi* Huang & Nel, 2009	Jurassic (Bathonian)	Inner Mongolia, China
*Juraembia ningchengensis* Huang & Nel, 2009	Jurassic (Bathonian)	Inner Mongolia, China

* This species has been placed in the genus “*Lithembia*” by Ross (1984) but as noted by Engel and Grimaldi (2006) and Miller (2009) the generic name is a *nomen nudum* and so we have reverted to Cockerell’s original combination for our table. The species very likely does not belong to *Embia* and the two syntypes (UCM-4421 and YPM-26169) should be re-examined and critically revised (based on photographs of the specimens they would appear to have the primitive condition of basal vein branching as delimited by Szumik (1996) (Engel pers. obs.).** E.S. Ross (pers. comm. 2010) presently does not consider these to belong to Embiodea and, indeed, the presence of a distinct ovipositor, fully-winged females, absence of probasitarsal modifications (which is not swollen despite the assertion of the authors), absence of a radial blood sinus (indeed, from the figures provided, the presence of any blood sinuses seems to require confirmation), and cerci with three cercomeres exclude the species from the order. These species certainly require revision, as do all compressions presently assigned to Embiodea.

## Material and methods

The age, origin, and biotic diversity of the Cambay amber are reviewed by Rust et al. (2010). Briefly, the amber occurs in rich concentrations within lignite mines in Gujurat State, western India. Its dating based on microfossils is earliest Eocene, ca. 50–52 Ma, just prior to complete suturing of India to the Asian continental plate. The amber was formed by trees in the Dipterocarpaceae, which are the dominant rainforest trees in Southeast Asia today. Specimens were prepared and preserved using the methods described by Nascimbene and Silverstein (2000). Morphological terminology and abbreviations largely follow those of Ross (2000) and the general classification is modified from (Ross (1970, 2001, 2003a, 2003b, 2006, 2007), (Szumik (1996, 2004), Engel and Grimaldi (2006), Szumik et al. (2008), and Miller (2009). Measurements were made using an ocular micrometer on an Olympus SZX-12 stereomicroscope and photomicrographs prepared using a Nikon D1× digital camera attached to an Infinity K-2 long-distance microscope lens.

## Systematic Paleontology

### Family Scelembiidae

Ross, 2001

http://species-id.net/wiki/Scelembiidae

#### Diagnosis.

Mandibles depressed, with incisive teeth well differentiated from molar area. Wings without crossveins between MA_1_ and MA_2_; CuA frequently diffuse. Male 10T with a membranous area occupying base and center of sclerite; 10R and 10L connected by thin basal bar; 10RP_2_ present, short, thumb-like; 10LP_1_ a curved, apically-forked process; HP rectangular, centered; LC_1_ with setae on apical area.

#### Included genera.

*Ambonembia* Ross (=*Ischnosembia* Ross), *Biguembia* Szumik (=*Aphanembia* Ross), *Gibocercus* Szumik, *Kumarembia* Engel and Grimaldi gen. n., *Litosembia* Ross, *Malacosembia* Ross, *Navasiella* Davis, *Pararhagadochir* Davis, and *Rhagadochir* Enderlein (=*Scelembia* Ross) (Szumik 2004).

### Kumarembia

Engel & Grimaldi gen. n.

urn:lsid:zoobank.org:act:F30F7AFB-D379-4F74-A76F-612169FD3B48

http://species-id.net/wiki/Kumarembia

#### Type species.

*Kumarembia hurleyi* Engel and Grimaldi, sp. n.

#### Diagnosis.

**Figure 3. F3:**
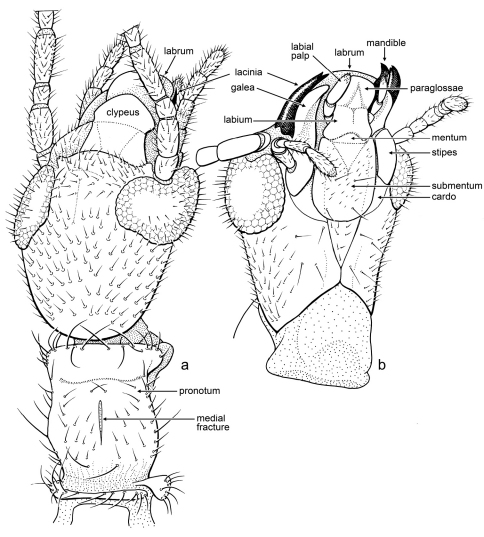
Line drawings of *Kumarembia hurleyi* Engel & Grimaldi, gen. et sp. n. **A** Head of holotype, dorsal view **B** Head of paratype, ventral view. Head length (to apex of labrum) 1.1 mm.

**Figure 4.**
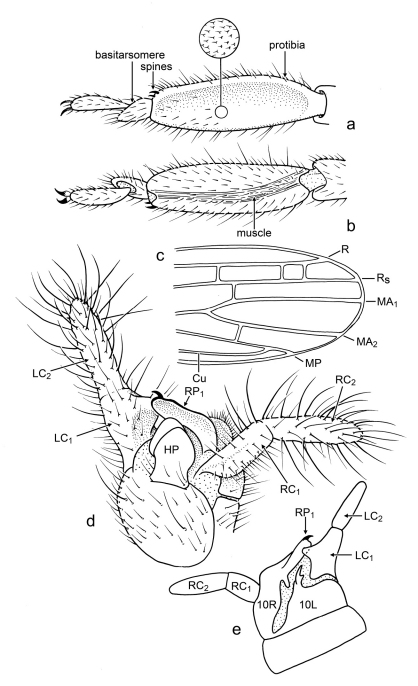
Line drawings of *Kumarembia hurleyi* Engel & Grimaldi, gen. et sp. n. (a, b, and d to same scale). **A** Protarsus of holotype, dorsal view **B** Protarsus of holotype, ventral view **C** Forewing apex of holotype **D** Male genitalia of holotype, ventral view **E** Male genitalia of holotype, dorsal view. Refer to description for individual measurements.

*Male*: Head relatively slender, longer than wide, elongate oval (Fig. 3a), slightly narrowed posteriad; compound eyes well developed, prominent, emarginate at base of antenna, and less so on posterodorsal margin, setose, with stiff setae, some longer than diameter of facets, and most setae on outermost distal surface (Fig. 3b); ocelli absent; antennae long, with 17 articles (incomplete in holotype, number of articles based on paratype), articles apparently uniformly sclerotized and pigmented (apical articles not differently pigmented or unpigmented); lacinia entirely sclerotized, with two small apical teeth (Fig. 3b), remainder of maxilla generalized; mentum sclerotized, small, approximately one-third length of labium, without setae, tightly joined to submentum; submentum sclerotic, with four stiff, very fine setae, anterior margin straight (appearing to have medial hump owing to mentum), lateral margins relatively straight and converging posteriorly toward base, margins meet before ventral margin of head capsule (Fig. 3b); ventral surface of head capsule, lateral to prementum, with eight (four pairs) fine, stiff, erect setae, head capsule otherwise with numerous decumbent setae, especially dorsally. Cervical area extensively membranous, especially ventrally. Pronotum longer than wide, well sclerotized and apparently pigmented, anterior margin straight, with prominent anterolateral corners, faintly constricted just posterior to anterior margin, posterior margin constricted, with rounded posterolateral corners, dorsally depressed just posterior to anterior margin (resulting in the anterior margin appearing somewhat lip-like), with thin, longitudinal, membranous “fracture” at midline (Fig. 3a). Wings large, mildly infumate; R reaching wing margin, straight apically (not procurved to terminate anteriorly); no c-r crossveins evident; Rs simple, terminating at wing apex (Fig. 4c), several r-rs crossveins present; single rs-ma_1_ crossvein present shortly after origin of MA_1_; MA apically forked, MA_1_ and MA_2_ both reaching wing margin, without crossveins between them (Fig. 4c); MP simple, reaching to apical wing margin; CuA apparently joining MP apically. Protibia greatest width 0.33× length, silk-producing surface slightly concave (Fig. 4a); distal end with two minute, sclerotized, slightly-curved, spine-like setae on mesal surface (Fig. 4a); metafemur swollen; metabasitarus (= metatarsomere I) elongate, without plantunlae (as in *Pararhagadochir*); metatarsomere II exceptionally short, without plantula; metadistitarsus (= metatarsomere III) elongate, nearly as long as combined lengths of metabasitarsus and metatarsomere II; pretarsal claws simple; arolium absent (Fig. 4a, 4b). Male terminalia asymmetrical; dorsally with left hemitergite (10L) relatively broad; right hemitergite (10R) relatively narrow, tapering posteriorly; hemitergites separated by membranous area, connected proximally by a thin sclerotic band (Fig. 4e); left tergal process (LP) sclerotized, short, curved, with forked apex, internal (caudad) hook longer than external (proximad) hook, both with tapered and pointed apices (Fig. 4e); right tergal caudal process (RP_1_) long, extending to LC_1_, apex with minute hook caudally and gentle lobe proximally; right tergal anterior process (RP_2_) present, short, thumb-like; ventrally with hypandrium (H) relatively large, broad, with rectangular hypandrial process (HP) positioned medially (Fig. 4d); cercomeres well sclerotized and uniformly covered by stiff, elongate setae in loose whorls (Fig. 4d); apical cercomeres (LC_2_ and RC_2_) slightly longer than basal cercomeres (similar to *Archembia*); left basal cercomere (LC_1_) medially expanded and lobed (Fig. 4d), left cercal basipodite comprising a sclerotic flange fused to outer rim of LC_1_ and without evidence of inner lobe or ring; right basal cercomere (RC_1_) well sclerotized throughout, cylindrical (Fig. 4d, 4e).

*Female*: Unknown.

#### Etymology.

The new generic name is a combination of Kumar (honoring Dr. Kumar Krishna, faithful colleague and dear friend, as well as the world’s leading authority on the systematics of Isoptera), and *Embia*, type genus of and frequent stem for embiodeans. The name is feminine.

### Kumarembia hurleyi

Engel & Grimaldi sp. n.

urn:lsid:zoobank.org:act:0124F7C1-BB06-4024-8C0A-3CFA053DD4CB

http://species-id.net/wiki/Kumarembia_hurleyi

Fig. 1 [Fig F2] [Fig F3] –4

#### Holotype.

♂; AMNH Tad-261-A (Fig. 1), India: Gujurat: Tadkeshwar lignite mine, Cambay Formation (Paleo-Eocene), 21°21.400'N, 73°4.532'E, 17–22 January 2010; to be deposited in the Birbal Sahni Institute of Paleobotany, Lucknow, India.

#### Paratype.

♂; AMNH Tad-253 (Fig. 2), India: Gujurat: Tadkeshwar lignite mine, Cambay Formation (Paleo-Eocene), 21°21.400'N, 73°4.532'E, 17–22 January 2010; in the Division of Invertebrate Zoology, American Museum of Natural History, New York.

#### Diagnosis.

As for the genus (*vide supra*).

#### Description.

*Male*: Total length (excluding wings and antennae, as preserved) 5.3 mm; forewing length (estimated) 5.1 mm, width 1.1 mm; integument generally light brown except darker on head and antenna, finely imbricate and impunctate where evident (based on paratype, integument of holotype slightly wrinkled owing to apparent desiccation and shrinkage of individual). Head length (to apex of labrum) 1.1 mm, width (just posterior to compound eyes) 0.64 mm, head posterior to compound eyes longer than compound eye diameter, posterior border gently rounded, covered with numerous, short, prominent setae, longer ventrally (Fig. 3). Pronotum length 0.56 mm, width (medial) 0.40 mm, apparently with weak longitudinal strigae in posterior half, with abundant fine setae as follows: anterior margin with row of ~10 setae, medial pair cruciate, lateral to these an upright pair, and lateral to those three pairs medioclinate setae; lateral margins with row ~8 erect setae of variable lengths; dorsal surface with two lateral rows of five short setae each; a short, anteromedial, cruciate pair, and longer posteromedial pair (Fig. 3). Wing membranes micronodulose and with numerous minute setae. LC_1_ length 0.28 mm, width at level of medial lobe 0.24 mm; LC_2_ length 0.36 mm; RC_1_ length 0.26 mm, RC_2_ length 0.35 mm.

*Female*: Unknown.

#### Etymology.

The specific epithet is a patronym honoring Mr. Ailan Hurley-Echevarria for his diligent efforts in processing and screening amber, during which he personally found one of the two specimens.

## Discussion

The phylogeny of most webspinner lineages remain contentious and in a state of flux. More importantly, numerous undescribed genera and species are known in collections and will likely have a strong influence on any estimations of relationship. It is therefore challenging to make fine determinations of the closest relatives for the Cambay amber fossils. *Kumarembia* can be placed within the Archembioidea clade by the 10T with a membranous area occupying the base and center of the sclerite, 10R and 10L connected by a thin basal bar, and 10RP_2_ present, short, and thumb-like. The genus can be placed within the Scelembiidae [= Group C of Szumik (2004); Group A = Archembiidae s.str., Group B = Pachylembiidae] by the rectangular and centered HP and the shape of 10LP_1_ which is a curved process and apically forked (simple, curved, and externally laminate in Pachylembiidae). This is quite significant given that other members of the clade occur in sub-Saharan Africa (Angola, Congo, Tanzania, Uganda) or in South America, particularly southern South America (e.g., Argentina, Bolivia, Brazil, and Peru, although *Pararhagadochir* is more widespread, extending as far north as Colombia and Venezuela). Accordingly, the discovery of a scelembiid in Cambay amber appears to represent one of the only Gondwanan elements of the fauna, while most other taxa show considerably different biogeographic affinities (Rust et al. 2010). As noted, relationships within Embiodea are contentious, with considerable cladistic inquiry revising phylogenetic hypotheses (e.g., Szumik 2004; Szumik et al. 2008; Klass and Ulbricht 2009). As these hypotheses of relationship continue to stabilize it will be interesting to explore further and refine the specific affinity of *Kumarembia cambayensis* with particular clades within Scelembiidae.

## Supplementary Material

XML Treatment for Scelembiidae

XML Treatment for Kumarembia

XML Treatment for Kumarembia hurleyi
